# Model Construction for the Intention to Use Telecare in Patients with Chronic Diseases

**DOI:** 10.1155/2013/650238

**Published:** 2013-03-05

**Authors:** Jui-Chen Huang, Yii-Ching Lee

**Affiliations:** ^1^Department of Health Business Administration, Hungkuang University, Taichung City 43302, Taiwan; ^2^No. 1018, Sector 6, Taiwan Boulevard, Shalu District, Taichung City 43302, Taiwan; ^3^Cheng Ching General Hospital, Taichung City 407, Taiwan

## Abstract

*Objective*. This study chose patients with chronic diseases as study subjects to investigate their intention to use telecare. *Methods*. A large medical institute in Taiwan was used as the sample unit. Patients older than 20 years, who had chronic diseases, were sampled by convenience sampling and surveyed with a structural questionnaire, and a total of 500 valid questionnaires were collected. Model construction was based on the Health Belief Model. The reliability and validity of the measurement model were tested using confirmatory factor analysis (CFA), and the causal model was explained by structural equation modeling (SEM). *Results*. The priority should be on promoting the perceived benefits of telecare, with a secondary focus on the external cues to action, such as promoting the influences of important people on the patients. *Conclusion*. The findings demonstrated that patients with chronic diseases use telecare differently from the general public. To promote the use and acceptance of telecare in patients with chronic diseases, technology developers should prioritize the promotion of the usefulness of telecare. In addition, policy makers can strengthen the marketing from media and medical personnel, in order to increase the acceptance of telecare by patients with chronic diseases.

## 1. Introduction

With the rapid aging of the population and the abrupt increase in the number of people needing care, the ability of a family to provide care has become relatively insufficient. Nowadays, the nuclear family is unable to support the care for family members, and the employment rate for women, who are traditionally the caretakers, is growing. As there lacks the understanding of telecare, and the government has limited funding to invest in related services, the amount of manpower providing care cannot meet the demands of the market.

Telecare refers to the utilization of distance communication techniques that link the user end and the service end to provide continuous, instant, and accessible care services. Telecare can be used not only in disease monitoring but also in health promotion and disease prevention. Its main clinical applications are in patients with chronic diseases, as well as the elderly or young patients. It has been shown to be especially effective in helping patients with chronic diseases (e.g., diabetes, coronary heart disease, and asthma) [1–3]. Although related industries are aggressively seeking cooperation with medical care industries and institutions, the technical maturity of related products needs to be continuously improved. In addition, the patients' needs must be taken into consideration during the design of practical products in order to increase the rate of telecare use.

### 1.1. Health Belief Model (HBM)

**Figure 1:**
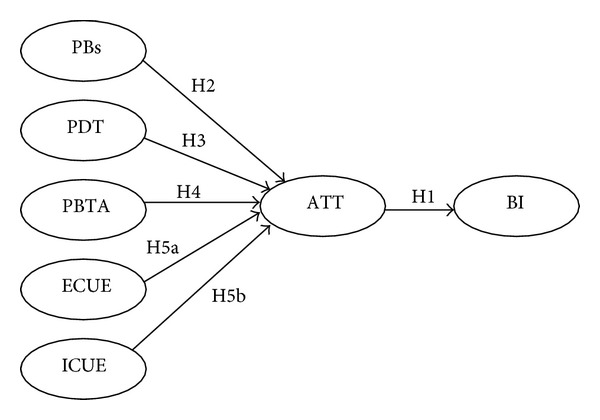
Research structure. Note: perceived benefits (PBs), perceived disease threat (PDT), perceived barriers of taking action (PBTA), external cues to action (ECUE), internal cues to action (ICUE), attitude toward using (ATT), and behavioral intention to use (BI).

The Health Belief Model (Figure 1) [4, 5] focuses on the four components, which are as follows:

1. perceived benefits (PBs): it refers to an individual's perception of the benefits gained from reducing disease risk and other unhealthy status;
2. perceived disease threats (PDTs): it refers to an individual's subjective perception of the possibility of having a specific disease and the severity of the influence of the disease on the individual;
3. perceived barriers of taking action (PBTA): it refers to an individual's perception of the negative influences created by taking the action of caring for the individual, such as high cost, inconvenience, time consumption, bodily pain, and side effects;
4. the individual's cues to actions (CUES): it refers to the process change due to the introduction of certain factors such as the internal factor of physiological condition and the external factors of mass media education, advice from friends and relatives, and the urging of medical professionals for the implementation of behaviors that promote health [6].

HBM is commonly used in health behavior research and is applicable in explaining or predicting health related behaviors [7, 8]. However, HBM has been rarely applied in telecare-related researches. Therefore, this study used HBM to understand the intention to use telecare in patients with chronic diseases. A large medical institute in Taiwan was used as an example, and the findings could provide an important reference for future managers, policy makers, and researchers in related fields.

Many theoretical and empirical studies have shown that there is a strong and significant causal relationship between the behavioral intention (BI) to use and the use of technology. Moreover, it has been found that intention predicts the actual use of technology [9, 10]. Based on Agarwal and Prasad's [9] argument (that behavioral intention is more appropriate than actual use as a measure of belief), this study used BI rather than actual use, because telecare in Taiwan is in its early stage, and there are very few actual users available for study. BI was, therefore, considered an independent measure that was more in line with actual needs.

This study proposed the following hypotheses (1 to 5) based on HBM related researches [4, 5, 7, 8].

(H1) There is a positive correlation between individuals' attitudes toward using (ATT) and the behavioral intention to use telecare.
(H2) Individuals with higher perceived benefits of telecare will have a higher positive attitude to use telecare.
(H3) Individuals with higher perceived disease threats (i.e., higher perceived susceptibility and severity) will have a higher positive attitude to use telecare.
(H4) Individuals with higher perceived barriers of taking action will have a lower positive attitude to use telecare.
(H5) Individuals with higher cues to action will have a higher positive attitude to use telecare.
  (H5a) Individuals with higher external cues to action (ECUES) will have a higher positive attitude to use telecare.
  (H5b) Individuals with higher internal cues to action (ICUES) will have a higher positive attitude to use telecare.

## 2. Materials and Methods

### 2.1. Research Design and Data Collection

This study chose patients with chronic diseases as study subjects, in order to respond aggressively to the pressing issues of a rapid aging population, including the abrupt increase in the percentage of the population needing care, insufficient governmental funding and manpower preparation, the long-term occupation of hospital beds by patients with chronic diseases, the rising cost of hospice care, and the need to promote life quality in patients with chronic diseases. This study investigated the intention of patients with chronic diseases to use telecare. A large medical institute in Taiwan was used as the sample unit. Patients older than 20 years who had chronic diseases (such as diabetes, heart disease, and asthma) were surveyed using convenience sampling and a structural questionnaire, and a total of 500 valid questionnaires were collected. Of the respondents, 55.8% are females, and most are aged 45 to 54 years old (20.8%), followed by above 65 years old and 35 to 44 years old (19.8% and 17.4%, resp.). Most of the respondents are high school educated (29.1%) followed by a college education level (28.9%). Most of the respondents' monthly incomes are 20,001 ~ 50,000 NTD (37.2%) followed by 50,001 ~ 80,000 NTD (29.3%) (US$1 ≈ NT$29.54).

### 2.2. Measures of the Constructs

This study was based on the definition and the constructs related to HBM [4, 5]. HBM is comprised of seven constructs: PB, PDT, PBTA, ECUE, ICUE, ATT, and BI. The operationalization and sources of the measurement items used in this study are shown in Table 1. The evaluation items employed a five-point Likert-type scale for measurement, ranging from 1 (strongly disagree) to 5 (strongly agree).

### 2.3. Data Analysis Methods

Confirmatory factor analysis (CFA) was employed to examine the reliability and validity of the measurement model. Furthermore, the structural equation modeling (SEM) technique was employed to interpret the causal model, and LISREL 8.7 was used for information analysis. Each of the impact coefficients was estimated using the maximum likelihood estimates method. The model's overall appropriateness of fit was evaluated using the following indicators: goodness of fit index (GFI), adjusted goodness of fit index (AGFI), normalized fit index (NFI), non-normed fit index (NNFI), incremental fit index (IFI), relative fit index (RFI), comparative fit index (CFI), parsimony goodness of fit index (PGFI), root mean square residual (RMR), and the root mean square error of approximation (RMSEA).

## 3. Results

### 3.1. Validity and Reliability of Measurement Model

In this study, the measurement model included 23 indicators describing seven latent constructs (PB, PDT, PBTA, ECUE, ICUE, ATT, and BI). As suggested by Hu and Bentler [12], the overall model fit was assessed using ten goodness-of-fit indices: GFI, AGFI, NFI, NNFI, IFI, RFI, CFI, PGFI, RMR, and RMSEA.  Table 2 shows the results of the confirmatory factor analysis (CFA). Eight fit indicators (NFI, NNFI, RFI, IFI, CFI, PGFI, RMSEA, and RMR) all reached the suggested standards, and the other fit indicators (GFI and AGFI) were slightly lower than the required standards (0.81 and 0.75, resp.). The structural equation analysis (SEM) by Baumgartner and Homburg [13] of 184 papers published between 1977 to 1994 in four journals (i.e., the Journal of Marketing, Journal of Marketing Research, International Journal of Research in Marketing, and Journal of Consumer Research) showed that 24% and 48% of the published papers reported that GFI and AGFI fit indicators lower than the suggested standards could still be considered as acceptable. Therefore, this study concluded that the fit indicators of this study were within an acceptable range.

The reliability and validity of the measurement model were examined using confirmatory factor analysis (CFA). Cronbach's alpha value and the construct reliability value were used to measure the internal consistency of the measurement model (see Table 3). The construct reliability was between 0.853 ~ 0.990, and Cronbach's alpha was between 0.851 ~ 0.990. Both were higher than the standard of 0.6 suggested by Bagozzi and Yi [14]. The results showed a high internal consistency of the research variables and indicated the good reliability of the latent variables used in this research. 

Construct validity includes convergent validity and discriminant validity. Convergent validity refers to the high degree of correlation of different measurement methods used to measure the same dimension. The standardized factor loading of all measurement variables in this study was between 0.67 ~ 0.98 (see Table 3), which was all above the suggested value of 0.5 [15] and indicated good convergent validity of the measurement model in this study.

Discriminant validity refers to a low degree of correlation of the measurement methods (either different or the same) used to measure two different dimensions. The chi-square difference test was used to measure the change in the chi-square value assuming the correlation of two dimensions as 1. The results showed that the chi-square value Δ*χ* ^2^ was higher than the critical value of 3.84, indicating good discriminant validity for the measurement tools.

### 3.2. Structural Model

The causal model was explained using structural equation modeling (SEM).  Table 2 shows the model fit indicators. After comparing all the fit indicators with the suggested standards, this study concluded that the fit indicators of the structural model were good.

**Figure 2:**
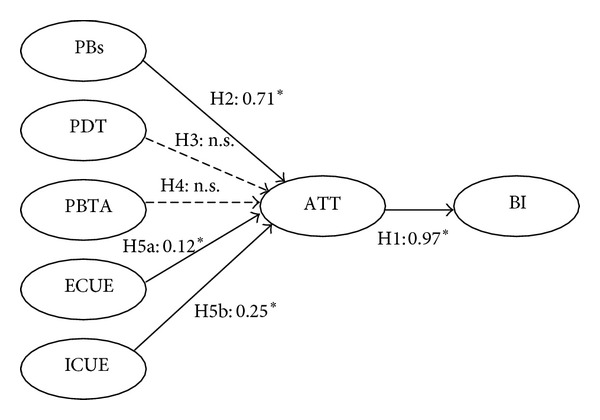
The results of structural model. Note: perceived benefits (PBs), perceived disease threat (PDT), perceived barriers of taking action (PBTA), external cues to action (ECUE), internal cues to action (ICUE), attitude toward using (ATT), behavioral intention to use (BI). *Path coefficient is significant at the 0.05 level. n.s. insignificant at the 0.05 level (path coefficient is obtained from this study).

In addition, hypotheses 1, 2, 5a, and 5b were supported based on the structural model shown in Figure 2. Specifically, the attitude toward using (ATT) was found to positively influence the behavioral intention to use (BI) significantly (the standardized path coefficient of (H1) = 0.97), while the perceived benefits (PBs) were found to positively influence the attitude toward using (ATT) significantly (the standardized path coefficient of (H2) = 0.71). External cues to action (ECUE) positively and significantly influences attitude toward using (ATT) (the standardized path coefficient of (H5a) = 0.12), and internal cues to action (ICUE) positively and significantly influences attitude toward using (ATT) (the standardized path coefficient of (H5b) = 0.25).

Neither the perceived disease threat (PDT) nor the perceived barriers to taking action (PBTA) had a statistically significant influence on attitude toward using (ATT); therefore, hypotheses 1, 2, 5a, and 5b were supported, while hypotheses 3 and 4 were not supported by the analysis.

Furthermore, the *R* ^2^ value for behavioral intention to use (BI) was 0.972, which was larger than 0.5, indicating that a high degree of variation could be explained by the model. 

## 4. Discussion

This study aimed to use HBM to predict the intention to use telecare in patients with chronic diseases. Structural equation modeling showed that the perceived benefits (PBs), external cues to action (ECUS), and internal cues to action (ICUE) all had significant and positive influences on the attitude toward using (ATT) telecare in patients with chronic diseases. The highest influence came from PB, followed by ICUE, and ECUE. The results were consistent with related researches that found PB having a positive and significant influence on ATT [4, 5, 12, 16] but were different from Huang and Lin [16] results, in which ICUE and ECUE did not have a significant level of influence on ATT. This study concluded that while promoting the use of telecare, priority may be given to promotion of the users' perceived benefits (PBs).

Furthermore, this study did not find significant influences of the perceived disease threat (PDT) and perceived barriers of taking action (PBTA) on the attitude toward using (ATT). This result was different from the conclusions of other studies adopting HBM, in which the general findings showed a significant positive influence of PDT on behavioral intention [4, 5, 16, 17] and a significant negative influence of PBTA on ATT [4, 5, 16]. In addition, this study found a significant positive correlation between attitude toward using and the behavioral intention to use telecare. This result was consistent with previous findings in related researches [9, 18–21].

In summary, this study found that the attitude toward using should be strengthened and that the priority should be placed on promoting the patients' perceived benefits, in order to promote the behavioral intention to use telecare in patients with chronic diseases. The patients' PB could be promoted by strengthening the health monitoring functions of telecare, thereby increasing the convenience of receiving medical care through the use of telecare, making the patients feel safer, and giving them an elevated quality of life. Another focus could be on providing external cues to action, such as increasing the influence of important people (medical personnel, media, relatives, friends, and family members). In doing so, the multiple advantages of telecare could be reached in regards to the welfare of patients with chronic diseases.

## 5. Conclusions

Few studies have used HBM to investigate the intention to use telecare. Huang and Lin [16] investigated the intention of the general public to use telecare. Their results differed from the findings in this study, which found that the perceived benefits were the major factor influencing the attitude toward using, followed by the perceived barriers of taking action (a significantly negative correlation), and the perceived disease threat (a significantly positive correlation). Neither external nor internal cues to action reached statistical significance. Based on the differences in the results of this study and that of Huang and Lin [16], the factors influencing the intention to use telecare may be different for the general public and for patients with chronic diseases. The perceived benefits are the major influencing factor for both populations; however, in patients with chronic diseases, internal and external cues to action have higher influences, while in the general public, the perceived barriers of taking action and the perceived disease threat are more influential. Therefore, based on the findings of this study, it was proposed that different methods should be used to promote the attitude toward using and the actual usage of telecare, according to the different demographic characteristics of the subjects. When promoting the use of telecare in patients with chronic diseases, priority should be placed on the perceived benefits, with a secondary focus on external cues to action. The findings of this study could provide an important reference for future related research.

This study had a number of conclusions. First, the findings demonstrated that patients with chronic diseases use telecare differently from the general public. The main contribution of this study was to explore the intention of patients with chronic diseases to use telecare. Second, different methods should be used to promote the attitude toward using and the actual usage of telecare according to the different demographic characteristics of the subjects. To promote the use of telecare in patients with chronic diseases, priority should be placed on promoting the perceived benefits, with a secondary focus on external cues to action, such as promoting the influences of important people on the patients (medical personnel, media, family members, relatives, and friends). Third, when promoting the use and acceptance of telecare in patients with chronic diseases, technology developers should prioritize the promotion of the usefulness of telecare. Policy makers can also strengthen the marketing of media and medical personnel to increase the acceptance of telecare by patients with chronic diseases. This approach could be used as an important reference for policy makers to use in promoting telecare for patients with chronic diseases and as a reference for related healthcare information technology industries.

In addition, there were several benefits of this study. First, the results could assist telecare related technological industries in their further development and in the enhancement of their competitiveness to develop products meeting the patients' needs. Second, the results could assist in the understanding of the predictions for the use of telecare by patients with chronic diseases, in order to avoid the waste of medical resources and to suppress rising health care costs.

The results of this study have great reference value for Taiwan, where telecare development is still at its early stage. This study could provide references pertaining to the predictions of important variables influencing the use of telecare and the relationship among these variables for researchers in related fields, technology developers, and policy makers [22–24].

## Figures and Tables

**Table 1:** Reliability and validity results.

Categories	Measure
Perceived benefits (PBs) [4, 5]	
PB1	I find that using telecare is helpful in monitoring health.
PB2	I find that using telecare makes me safer in my daily life.
PB3	Telecare can enhance my level of convenience in accessing medical care service.
PB4	Telecare can enhance the quality of my life.
Perceived disease threat (PDT) [4, 5]	
PDT1	I find that I can fall ill easier than others.
PDT2	I find that I can suffer from high blood pressure, diabetes, heart disease, and other chronic diseases in the future.
PDT3	I find that my health is deteriorating.
PDT4	I find that I can suffer from high blood pressure, diabetes, heart disease, and other chronic diseases in the future and could be forced to change my previous way of life.
Perceived barriers of taking action (PBTA) [4, 5]	
PBTA1	I am concerned that telecare is not adequately secure and that it might lead to the leak or abuse of my personal information.
PBTA2	I am concerned that telecare would violate my privacy.
PBTA3	I am concerned that the accuracy and reliability of the instruments of telecare are not high enough.
External cues to action (ECUE) [4, 5]	
ECUE1	Relatives encourage and support me to use telecare.
ECUE2	Friends encourage and support me to use telecare.
ECUE3	Medical care personnel encourage and support me to use telecare.
ECUE4	Media endorses the use of telecare.
Internal cues to action (ICUE) [4, 5]	
ICUE1	How many times did you fall sick in the last three months?
Attitude toward using (ATT) [11]	
ATT1	I like using telecare.
ATT2	Overall, I consider telecare to be just right.
ATT3	In my old age, using telecare would be ideal.
Behavioral intention to use (BI) [11]	
BI1	Overall, I am highly willing to use telecare.
BI2	If necessary, I would use telecare often.
BI3	In my old age, I am willing to use telecare.
BI4	In my old age, I would use telecare often.

**Table 2:** Fit indices for measurement and structural model.

Fit indices	Recommended value	Measurement model	Structural model
GFI	≥0.9	0.81	0.81
AGFI	≥0.8	0.75	0.76
NFI	≥0.9	0.94	0.93
NNFI	≥0.9	0.93	0.94
RFI	≥0.9	0.92	0.92
IFI	≥0.9	0.94	0.94
CFI	≥0.9	0.94	0.94
PGFI	≥0.5	0.62	0.64
RMSEA	≤0.1	0.1	0.1
RMR	≤0.05	0.05	0.05

**Table 3:** Reliability and validity results.

Items	Standardized factor loading
Perceived benefits (PBs) (0.946, 0.951)^a^	
PB1	0.95
PB2	0.97
PB3	0.84
PB4	0.83
Perceived disease threat (PDT) (0.853, 0.851)^a^	
PDT1	0.67
PDT2	0.85
PDT3	0.75
PDT4	0.80
Perceived barriers of taking action (PBTA) (0.897, 0.887)^a^	
PBTA1	0.91
PBTA2	0.98
PBTA3	0.68
External cues to action (ECUE) (0.907, 0.913)^a^	
ECUE1	0.95
ECUE2	0.97
ECUE3	0.75
ECUE4	0.67
Attitude toward using (ATT) (0.934, 0.941)^a^	
ATT1	0.83
ATT2	0.92
ATT3	0.97
Behavioral intention to use (BI) (0.990, 0.990)^a^	
BI1	0.98
BI2	0.98
BI3	0.98
BI4	0.98

^a^Values in parentheses for constructs indicate construct reliability and Cronbach's alpha, respectively.
